# Improved Stethescope, Sore Nipples, &c.

**Published:** 1855-03

**Authors:** 


*Improved Stethoscope.*—Dr. Camman. of New York City, has invented a new stethoscope, which intensifies, to an extraordinary degree, every sound heard in ausculation. This intensity is produced by both ears of the observer being acted upon at once, and the ear-pieces fitting tightly into the meatus of both ears, all external sounds are more thoroughly cut off, and the mind of the ascultator is thus forcibly drawn to the phenomena taking place within the thorax. We hope to be able to give a full description of this instrument in our next number.

*Sore Nipples.*—Dr. Bourdel, assistant professor at Montpellier, states in the *Gazette Medicale* of Toulouse, that for the last ten years he has used with advantage the simple tincture of benzoin in the soreness of the nipples occurring during lactation, whether the fissures were superficial or deep, large or small, recent or of long standing. The tincture is applied with a camel's hair brush, and forms a layer which protects the nipple from the contact of the air and clothing. The first application causes some pain. The child takes the breast without repugnance as soon as the liquid has dried. A cure may be anticipated in from three to twelve days.

*Vomiting.*—Dr. Lobach adds the weight of his extensive experience to the opinion which has been repeatedly advanced of late in regard to the utility of the tincture of nux vomica in cases of obstinate vomiting during pregnancy. The nux vomica is given in doses of four drops every two hours.

Never give Mercury to a debilitated patient, nor to one with a weakened state of the system.

Cut off the upper third of a Tonsil and the rest will dwindle away.

In inflammation, just before healthy termination takes place, a copious secretion from the inflamed part occurs, indicative of the impending resolution.

The abuse of mercury strongly predisposes the system to the morbific effects of a low and variable temperature.

Never leave a wound ragged.

